# Effects of low doses of methylmercury (MeHg) exposure on definitive endoderm cell differentiation in human embryonic stem cells

**DOI:** 10.1007/s00204-023-03580-7

**Published:** 2023-08-23

**Authors:** Bai Li, Xiaolei Jin, Hing Man Chan

**Affiliations:** 1 Department of Biology, University of Ottawa, 30 Marie Curie, Ottawa, ON K1N 6N5 Canada; 2 Regulatory Toxicology Research Division, Bureau of Chemical Safety, Food Directorate, HPFB, Health Canada, 251 Sir Frederick Banting Driveway, Ottawa, ON K1A 0K9 Canada

**Keywords:** Methylmercury, Developmental toxicity, Embryonic stem cell, Definitive endoderm, Adverse outcome pathway, Non-neuronal organs

## Abstract

Fetal development is one of the most sensitive windows to methylmercury (MeHg) toxicity. Laboratory and epidemiological studies have shown a dose–response relationship between fetal MeHg exposure and neuro performance in different life stages from infants to adults. In addition, MeHg exposure has been reported to be associated with disorders in endoderm-derived organs, such as morphological changes in liver cells and pancreatic cell dysfunctions. However, the mechanisms of the effects of MeHg on non-neuronal organs or systems, especially during the early development of endoderm-derived organs, remain unclear. Here we determined the effects of low concentrations of MeHg exposure during the differentiation of definitive endoderm (DE) cells from human embryonic stem cells (hESCs). hESCs were exposed to MeHg (0, 10, 100, and 200 nM) that covers the range of Hg concentrations typically found in human maternal blood during DE cell induction. Transcriptomic analysis showed that sub-lethal doses of MeHg exposure could alter global gene expression patterns during hESC to DE cell differentiation, leading to increased expression of endodermal genes/proteins and the over-promotion of endodermal fate, mainly through disrupting calcium homeostasis and generating ROS. Bioinformatic analysis results suggested that MeHg exerts its developmental toxicity mainly by disrupting ribosome biogenesis during early cell lineage differentiation. This disruption could lead to aberrant growth or dysfunctions of the developing endoderm-derived organs, and it may be the underlying mechanism for the observed congenital diseases later in life. Based on the results, we proposed an adverse outcome pathway for the effects of MeHg exposure during human embryonic stem cells to definitive endoderm differentiation.

**Supplementary Information:**

The online version contains supplementary material available at 10.1007/s00204-023-03580-7.

## Introduction

Mercury (Hg) is a well-recognized environmental contaminant that is of global concern (Driscoll et al. 2013). In the environment, Hg (Hg^0^) occurs in its elemental form, as inorganic salts (Hg^+^ or Hg^2+^), and as organic compounds such as methylmercury (MeHg) (Clarkson 2002). Anthropogenic effects have increased Hg emission dramatically into the environment (Streets et al. 2009), which is further converted to MeHg through anaerobic microorganisms, typically sulfur-reducing bacteria, in aquatic environments (Clarkson 2002; Parks et al. 2013). Methylmercury can then bioaccumulate along the aquatic food chains into top marine/freshwater predators, making communities or populations that rely heavily on fish consumption in their diets susceptible to MeHg exposure (Driscoll et al. 2013; Van Oostdam et al. 2005). Also, rice which grows in moist, anaerobic soil that is ideal for microbial methylation of mercury, can also accumulate MeHg, and affect the rice-consuming populations (Feng et al. 2021).

The main target site for MeHg is the central nervous system, with clinical findings showing MeHg-induced neurobehavioral and cognitive function impairments in the victims of the Japanese and Iraqi MeHg poisoning events (Amin‐Zaki et al. 1981; Bakir et al. 1973; Harada 1978, 1995). Animal studies under well-controlled laboratory conditions have also confirmed the neurotoxic effects of MeHg, with the developing central nervous systems being especially susceptible, causing more severe effects in prenatal exposure compared to exposure in later life stages (Castoldi et al. 2008). This is in part attributed to MeHg-induced negative effects on neuronal migration and differentiation (Bose et al. 2012; Fujimura and Usuki 2015; Tamm et al. 2006), and also MeHg-induced cell death due to distortion of intracellular calcium and glutamate homeostasis, oxidative stress generation, as well as mitochondrial dysfunction (dos Santos et al. 2016; Nagashima 1997).

In addition to its well-characterized neurotoxic effects, MeHg exposure can also lead to adverse effects in tissues other than the brain. In victims of the MeHg poisoning event in Japan, erosive inflammation of the digestive tract, fatty degeneration of the liver and kidney, and disturbance of pancreatic islet cells were all observed in addition to neuronal disturbances (Eto 1997; Eto et al. 2010; Harada 1995). Furthermore, the hepatic disorder was also identified as a cause of death for MeHg poisoning victims, with comparable levels of MeHg accumulation in the liver compared to the brain (Harada 1995). Multiple cohort studies have shown a positive correlation between mercury exposure levels and elevated insulin levels accompanied by decreased pancreatic β-cell function (Chang et al. 2011; He et al. 2013). Animal studies also have corroborated epidemiology findings, showing that MeHg accumulated within the liver to comparable levels of the brain (Carneiro et al. 2014; Gonzalez et al. 2005), resulting in imbalanced redox, hepatic glycogen accumulation, disruption of energy metabolism pathways, as well as alteration of global gene expression profiles within the liver (da Rosa-Silva et al. 2020; Mela et al. 2007; Ung et al. 2010; Yadetie et al. 2013). Apoptosis and dysfunction in pancreatic β-cells through oxidative stress were also observed (Chen et al. 2006; Schumacher and Abbott 2017; Yang et al. 2022). In vitro studies on non-brain derived cell lines exposed to MeHg, though limited, have shown that MeHg-induced oxidative stress resulted in apoptosis in liver-derived HepG2 cells (Cuello et al. 2010) and altered cell morphology in human-induced pluripotent stem-cell-derived hepatocytes (Jamalpoor et al. 2022). Despite these observations, the mechanism of the effects of MeHg exposure on non-brain-related organs is still poorly understood compared to the effects of MeHg on the brain, and studies examining the developmental effects of MeHg exposure on non-brain related organs is still lacking.

In recent years, there is a growing trend of reducing the use of animals for testing of hazardous compounds, and in vitro toxicity testing that relies on cultured cells has emerged as an alternative tool for toxicity assessment. Among these, embryonic stem cells, and especially human embryonic stem cells (hESCs), which have the potential to differentiate into all fetal cell lineages represent an attractive cellular system for in vitro studies in developmental toxicology (Visan et al. 2012), and has been utilized to examine the embryotoxicity of MeHg (Li et al. 2021; Seiler and Spielmann 2011; Stummann et al. 2007). As the effects of MeHg on neuronal differentiation have been extensively studied (He et al. 2012; Prince et al. 2021; Stummann et al. 2009; Tamm et al. 2008; Theunissen et al. 2011; Zimmer et al. 2011), our objective is to examine the developmental toxicity of MeHg on other non-neural-derived organs using hESCs as a model. Since the potentially affected organs such as the liver, pancreas, and the digestive tract (Eto 1997; Eto et al. 2010; Harada 1995) are endoderm derived, and the endoderm is the first germ layer to develop out of the three germ layers (Muhr and Ackerman 2020), we hypothesized that MeHg exposure affects the early differentiation of the endoderm in the hESCs. Using a transcriptomics approach, we aim to not only characterize the effects of MeHg during definitive endoderm formation but also explore the potential cellular mechanisms that lead to developmental toxicity due to MeHg exposure. We hypothesized that MeHg could disrupt the differentiation of endoderm formation in human stem cells by distortion of intracellular calcium and glutamate homeostasis, oxidative stress generation, as well as mitochondrial dysfunction.

## Materials and methods

### Ethics review and approval

This project was reviewed and approved by the Health Canada and Public Health Agency of Canada’s Research Ethics Board (File No. REB 2016-027H) and by the Office of Research Ethics and Integrity of the University of Ottawa (File No. H-05-19-4084).

### Cell maintenance and induction

Human embryonic stem cells (H9; passage 30; WiCell Research Institute) were maintained according to the protocols established in our previous study (Li et al. 2021). Briefly, hESCs were maintained and expanded in six-well plates, which were pre-coated with Matrigel (Catalog no. 354 230; BD Biosciences). Cells were cultured under 37 °C, 4% oxygen (O_2_) and 10% carbon dioxide (CO_2_) in a tri-gas incubator (Thermo Fisher Scientific) and fresh Essential 8 Flex Medium (Catalog no. A2858501; Thermo Fisher Scientific) was changed daily. On the first day of seeding, 10 µM Rho-associated kinase inhibitor (Catalog no. 72304; StemCell Technologies) was added to the medium to enhance the survival of the cells. Cells were gently detached with Gibco StemPro Accutase cell dissociation reagent (Catalog no. A1110501; Thermo Fisher Scientific) and seeded onto new plates for subsequent experiments when the confluency reached around 85%. Only hESCs under passage 40 (within 10 passages from purchase) were used to avoid any serious genetic instability associated with prolonged passaging.

The hESCs were seeded in six-well plates at a density of 2 × 10^4^ cells/mL with the growth medium (Essential 8 Flex Medium) and incubated at 37 °C, 4% oxygen (O_2_) and 10% carbon dioxide (CO_2_) with daily medium change. When the cell confluency reached around 20%–30%, the growth medium was replaced with the definitive endoderm induction medium A from Gibco^™^ PSC Definitive Endoderm Induction Kit (A3062601, Thermo Fisher Scientific). 24 h later, the culture medium was disposed of, and the definitive endoderm induction medium B was used for another 24 h of incubation. The hESC-derived DE cells were verified by immunostaining for both self-renewal (OCT4) and endodermal protein markers (SOX17 and CXCR4) (Fig. S1). In brief, the cells were washed twice with phosphate-buffered saline (PBS) after disposing the induction medium B and fixing with 4% paraformaldehyde (Santa Cruz Biotechnology). The cells were then blocked and permeabilized with 5% bovine serum albumin (BSA) diluted in PBS containing 0.3% Triton X-100 (Sigma-Aldrich). The cells were, respectively, incubated with rabbit anti-human OCT4 antibody (Catalog no. C30A3; Cell Signaling Technology), goat anti-human SOX17 antibody (Catalog no. AF1924; R&D systems), and rabbit anti-human CXCR4 antibody (Catalog no. ab124824; Abcam) diluted at 1:200 in 5% BSA, and incubated overnight at 4 °C. The cells were then gently washed with PBS for three times and, respectively, incubated with Alexa Fluor 488 goat anti-rabbit IgG (Catalog no. A11008; Thermo Fisher Scientific), and Alexa Fluor 594 donkey anti-goat IgG (Catalog no. A11058; Thermo Fisher Scientific) diluted at 1:500 in 5% BSA at 4 °C, overnight. Prolong Gold antifade reagent containing 4’,6-diamidino-2-phenylindole (DAPI; Catalog no. S36939; Thermo Fisher Scientific) was added and the immunofluorescent images were taken with a Nikon A1RsiMP confocal microscope (Nikon Instruments Inc.) with Nikon’s Imaging Software NIS-Elements.

### Chemical preparation and exposure

MeHg chloride (purity: 99.9%) was obtained from Sigma-Aldrich and dissolved in dimethyl sulfoxide (DMSO, Sigma) to obtain 1000 × stock solutions (i.e., 0.001, 0.01, 0.1, 0.2, 0.3, 0.4, 0.5, 1, 5 and 10 mM). hESCs were seeded in six-well plates and allowed for growth until they reached a confluency of around 20%–30%. The cells were then exposed to 0 (DMSO as vehicle control), 0.001, 0.01, 0.1, 0.2, 0.3, 0.4, 0.5, 1, 5 or 10 µM MeHg by 1000 × dilution of the stock solutions in induction medium for the initial cell viability assay. The experimental doses were narrowed down according to the results of cell viability and the cells were treated with 0 (DMSO as vehicle control), 10, 100 or 200 nM MeHg by 1000 × dilution of the stock solutions in induction medium for the rest of assays. The cells were repeatedly dosed each day throughout the induction process.

### Cell viability and morphology

Cell viability was determined by a Cell Counting Kit 8 (WST-8/CCK8) (Catalog no. ab228554, Abcam Inc.) according to the manufacturer’s instructions. In brief, hESCs were seeded, induced, and exposed to MeHg as described above. At the end of induction and exposure, 200 μL of WST-8 solution was added into each well on the six-well culture plate followed by 10 s of shaking and incubation at 37 °C for 1.5 h in the dark. The absorbance at 460 nm wavelength was read on a BioTek Cytation 3 Cell Imaging Multi-Mode Reader (Fisher Scientific). The effects of 0, 10, 100, and 200 nM MeHg on cell viability were also checked by cell counting. Briefly, hESCs were seeded, induced, and exposed to MeHg as described above. At the end of induction and exposure, the medium was disposed, and cells were gently lifted by StemPro Accutase cell dissociation reagent after rinsing by PBS. Then the cells were centrifuged and re-suspended in 1 ml of PBS and gently pipetted to generate a single-cell suspension. 10 µL of the cell suspension was taken for cell counting, and the cell numbers were counted with a Countess Automated Cell Counter (Invitrogen, Thermo Fisher Scientific) after 1:1 dilution of cell suspension in Trypan blue solution (Catalog no. T10282; ThermoFisher Scientific).

For morphology observations, hESCs were seeded, induced to DE, and treated with MeHg, as described above. The cell morphology was observed and imaged at each time point (before induction, during induction and after induction) with a 10 × objective on a Zeiss Axiovert 40 CFL inverted microscope (Carl Zeiss Microscopy LLC) with a SPOT RT3 digital camera and SPOT basic software (Diagnostic Instruments Inc.).

### RNA sample preparation and next-generation sequencing

MeHg doses used for RNA sequencing were chosen based on cell viability and morphology results, as well as a literature review on the Hg concentrations found in maternal blood (Table 1). The final doses of 0, 10, 100, and 200 nM of MeHg exposure were chosen for RNA sequencing as these doses did not induce significant cell death and covered the range of Hg concentrations typically found within human blood. hESCs were cultured, induced, and treated as described above. RNA samples were extracted and purified with a RNeasy Plus Mini kit (Catalog no. 74134, Qiagen) following the manufacturer’s protocol. In brief, the culture medium was completely removed, and cells were lysed directly with Buffer RLT Plus containing 10% β-mercaptoethanol (β-ME). The cell lysates were gently pipetted into microcentrifuge tubes and mixed by vortexing. Lysates were homogenized by centrifugation for 2 min at maximum speed (14,800 rpm) in QIAshredder spin columns placed in 2 ml collection tubes and were transferred to gDNA eliminator spin columns for gDNA removal. After centrifuging for 30 s at 10,000 rpm, 70% ethanol was added to the flow-through, and mixed well by pipetting. The mixed sample solutions were transferred to RNeasy spin columns and centrifuged for 15 s at 10,000 rpm. The flow-through was discarded, and spin column membranes were washed by centrifuging for 15 s at 10,000 rpm in Buffer RW1 and discarding the flow-through. The spin column membranes were then washed by centrifuging for 15 s at 10,000 rpm in Buffer RPE, followed by a second wash in Buffer RPE for 2 min at 10,000 rpm. The spin columns were carefully placed in new collection tubes and centrifuged for 1 min at maximum speed (14,800 rpm) to eliminate any possible residual Buffer RPE. To elute the RNA samples, the spin columns were placed in 1.5 mL collection tubes and 30 μL RNase-free water was added directly to the column membrane. The collection tubes were centrifuged for 1 min, and the eluted RNA samples were stored immediately at − 80 °C.

**Table 1:** Maternal blood concentrations of Hg in different populations

Population (Country)	Total Hg GM/AM/median (range), ug/L	Total Hg GM/AM/median (range), nM	MeHg GM/AM/median (range), ug/L	MeHg GM/AM/median (range), nM	References
Nunavik (Canada)	4.2 (3.4–4.9)	20.9 (16.9–24.4)			Furgal et al. (2018)
9 cities (Canada)	0.60	3.0			Lukina et al. (2021)
South Carolina (USA)	0.87 (0.02–3.9)	4.3 (0.1–19.4)	0.58 (0.01–2.7)	2.7 (0.05–12.5)	Donohue et al. (2018)
Madeira (Portugal)	9.0 (1.0–57.1)	44.9 (5.0–284.7)			Caetano et al. (2019)
3 provinces (Korea)	5.27 (5.0–5.57)	26.3 (24.9–27.8)	4.05 (3.81–4.32)	18.8 (17.7–20.0)	You et al. (2012)
Fifteen regional centers (Japan)	4.21 (0.334–30.1)	21.0 (1.7–150.1)			Kobayashi et al. (2019)
2 northeastern areas (Japan)	5.42 (0.61–25.19)	27.0 (3.0–125.6)	5.15 (0.6–24.99)	23.9 (2.8–115.9)	Iwai-Shimada et al. (2019)
Zhoushan (China)	5.68	28.3			Wu et al. (2014)
Guizhou (China)	3.0 (1.7–11)	15.0 (8.5–54.8)			Rothenberg et al. (2013)

The RNA samples were sent to Genome Quebec for quality check and whole genome-scale mRNA sequencing by Illumina NovaSeq 6000. Sequencing was performed by paired end reading with a sequencing depth of 50 M reads for each sample and three biological replicates for each treatment group.

### Sequencing data analysis and marker selection for verification

The raw data were obtained from Genome Quebec, and quality control was conducted with FastQC (https://www.bioinformatics.babraham.ac.uk/projects/fastqc/). All analyses were conducted in R (https://www.r-project.org/). Gene sequences were aligned to the reference human genome database GRCh38 (https://www.ncbi.nlm.nih.gov/data-hub/genome/GCF_000001405.26/), and reads counted using the “Rsubread” package (Liao et al. 2019). Data were then normalized, and lowly expressed genes were filtered out using the “edgeR” package (Robinson et al. 2010). Boxplots showed filtered data to reflect quantile normalization, and principal component analysis (PCA) was used to demonstrate the clustering of samples in different groups. The R package “edgeR” (Robinson et al. 2010) was used to identify differentially expressed genes (DEGs) with cutoff for DEGs set as adjusted *p* value < 0.05 and absolute log2 [fold change (FC)] > 1. Significant DEGs were further visualized in volcano plots and heat maps generated with the “ggplot2” package.

Gene set testing for DEGs was performed with the “clusterProfiler” package (Yu et al. 2012) for Gene Ontology (GO) functional enrichment and Kyoto Encyclopedia of Genes and Genomes (KEGG) pathway analysis. Adjusted *p* value < 0.05 and count ≥ 30 was set as the cutoff for GO functional enrichment, whereas adjusted *p* value < 0.05 was used as the cutoff for KEGG pathway analysis. Data were visualized using the “ggplot2” package.

GO functional enrichment analysis identified 32 GO terms that were significant, of which 7 were related to early development. A protein–protein interaction network analysis of the genes within each of these seven early development-related GO terms was conducted using Cytoscape (https://cytoscape.org/) to identify hub genes for each of these GO terms. Lineage genes classified based on the TaqMan hPSC scorecard assay (Tsankov et al. 2015) were also identified in the dataset, and a cross comparison of hub genes identified from GO terms and DEGs from lineage genes with genes driving endoderm differentiation in the literature (Aksoy et al. 2014; Chu et al. 2016; Faial et al. 2015; Teo et al. 2011; Xuan and Sussel 2016) narrowed down genes for further downstream qPCR and western blot verification.

We explored the potential mechanisms of the developmental toxicity of MeHg on hESCs while differentiating toward DE cells by identifying the sequence of responses to the increasing dose of MeHg. We entered the DEGs into a web-based tool, FastBMD (https://www.fastbmd.ca/) (Ewald et al. 2021), to calculate the benchmark doses (BMD). The DEGs were then ranked by their BMDs, and the enriched KEGG pathways were ranked based on the average rank of DEGs involved in each pathway (cutoff of involved DEG number ≥ 5).

### Reverse transcription quantitative polymerase chain reaction (RT-qPCR)

RNA samples were prepared as described above. RNA concentration was determined using NanoDrop^™^ 2000 spectrophotometer (Thermo Scientific) and RNA quality was verified using gel electrophoresis and qualitative assessment of the relative intensities of the 28 and 18 s ribosomal RNA bands. RNA was treated with DNaseI (Catalog no. 18068015, Invitrogen, ThermoFisher Scientific) prior to cDNA synthesis from 900 ng total RNA using a high-capacity cDNA reverse transcription kit (Catalog no. 4368814; ThermoFisher Scientific). Real-time PCR was performed in 20 µl reactions containing 10 µl SsoFast EvaGreen supermix (Catalog no. 1725201, Bio-Rad), 500 nM forward and reverse primers (Table S1), and 0.5 µl cDNA template obtained from cDNA synthesis. Controls (no template added) were run in every assay, and non-reverse transcribed samples were run for every primer pair. Reaction efficiency for all primer pairs was between 90 and 110%. Final data were normalized to the geometric mean of the three reference genes (*gapdh*, *hddc2* and *znf324b*) and the 0 nM group using the delta-delta CT method (Rao et al. 2013).

### Western blotting

To determine whether the effects of MeHg on gene expressions also occur at the protein level, we measured the protein expression of the selected markers by western blots.

hESCs were cultured, induced, and exposed to MeHg as described above. Culture medium was discarded, and the cells were washed with PBS and lysed with Pierce radioimmunoprecipitation assay buffer (RIPA buffer; Thermo Fisher Scientific) containing proteinase and phosphatase inhibitor cocktails (Catalog no. 5872S; Cell Signaling Technology). The cell lysates were then sonicated with a Microson Ultrasonic Cell Disruptor XL2000 (Misonix Inc.). The whole process of protein extraction was conducted on ice. Protein concentrations of the lysates were quantified with a Pierce Bicinchoninic Acid Protein Assay kit (Thermo Fisher Scientific). The cell lysates were then denatured by heating at 100 °C for 4 min after mixing with 4 × Laemmli sample buffer (Catalog no. 1610747; Bio-Rad).

Cell lysate samples were electrophoresed on 4–20% Mini-Protein TGX Stain-Free Precast Gels (Catalog no. 17000436, Bio-Rad). The same amount of protein was loaded for each batch, and the amount of total protein loaded ranged from 20 to 24 μg between different batches. Gels were then activated under ultraviolet light using the ChemiDoc XRS + imaging system (Bio-Rad), and proteins were electro-transferred from gels to polyvinylidene difluoride (PVDF) membranes with the Mini Trans-Blot Cell (Catalog no. 1703930; Bio-Rad). Membranes were then blocked with 5% non-fat dry milk (NFDM, Catalog no. 1706404; Bio-Rad) or 3% bovine serum albumin (BSA, Catalog no. BSA-50; Rockland Immunochemicals, Inc.) depending on the protein targets for 1 h at room temperature and incubated with antibodies of interest (Table S2) diluted in their corresponding blocking buffer at 4 °C overnight. Then the membranes were washed three times with Tris-buffered saline containing 0.1% Tween 20 (TBST) on a shaker for 5 min and incubated with 1:5,000 dilution of horseradish peroxidase (HRP)-linked secondary antibodies (Table S2) diluted in corresponding blocking buffer at 4 °C overnight. The membranes were rewashed for three times with TBST and incubated with enhanced chemiluminescence substrate (ECL, Catalog no. 1705062; Bio-Rad) in the dark and imaged with the ChemiDoc XRS + imaging system. The Image Lab software (Bio-Rad) was used to quantify the band intensities, and all target band intensities were normalized against the total protein band intensities.

As autophagy plays a critical role in the differentiation of stem cells (Sharma et al. 2022), the expression of autophagic protein markers (LC3B and SQSTM1, antibody information is shown in Table S2) was also measured by western blots as described above.

### Statistical analysis

All data were displayed as the means plus or minus the standard errors of the mean (means ± SEMs) and were tested for statistical significance with one-way ANOVA, followed by Dunnett’s post hoc tests. The differences were considered significant if *p* < 0.05. Except for the RNA sequencing data, which was analyzed with R as described above, all statistical analyses were performed using GraphPad Prism (version 8).

## Results

### Effects on morphology and cell viability of MeHg exposure during definitive endoderm (DE) differentiation

**Fig. 1:**
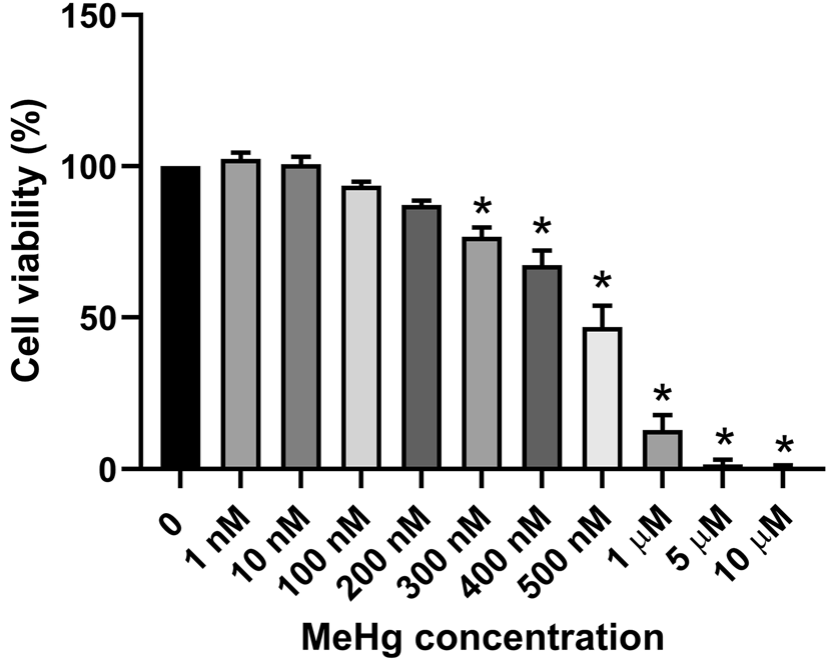
Cell viability (as percentage of control) of definitive endoderm (DE) cells differentiated from human embryonic stem cells (hESCs) exposed to 1 nM to 10 µM MeHg during DE differentiation. One-way ANOVA and Dunnett’s post hoc test were used at each time point to compare differences between treatment and 0 nM group with *p* < 0.05 being considered as significant. Data are presented as means ± SEMs with *N* = 6–9 for each group

**Fig. 2:**
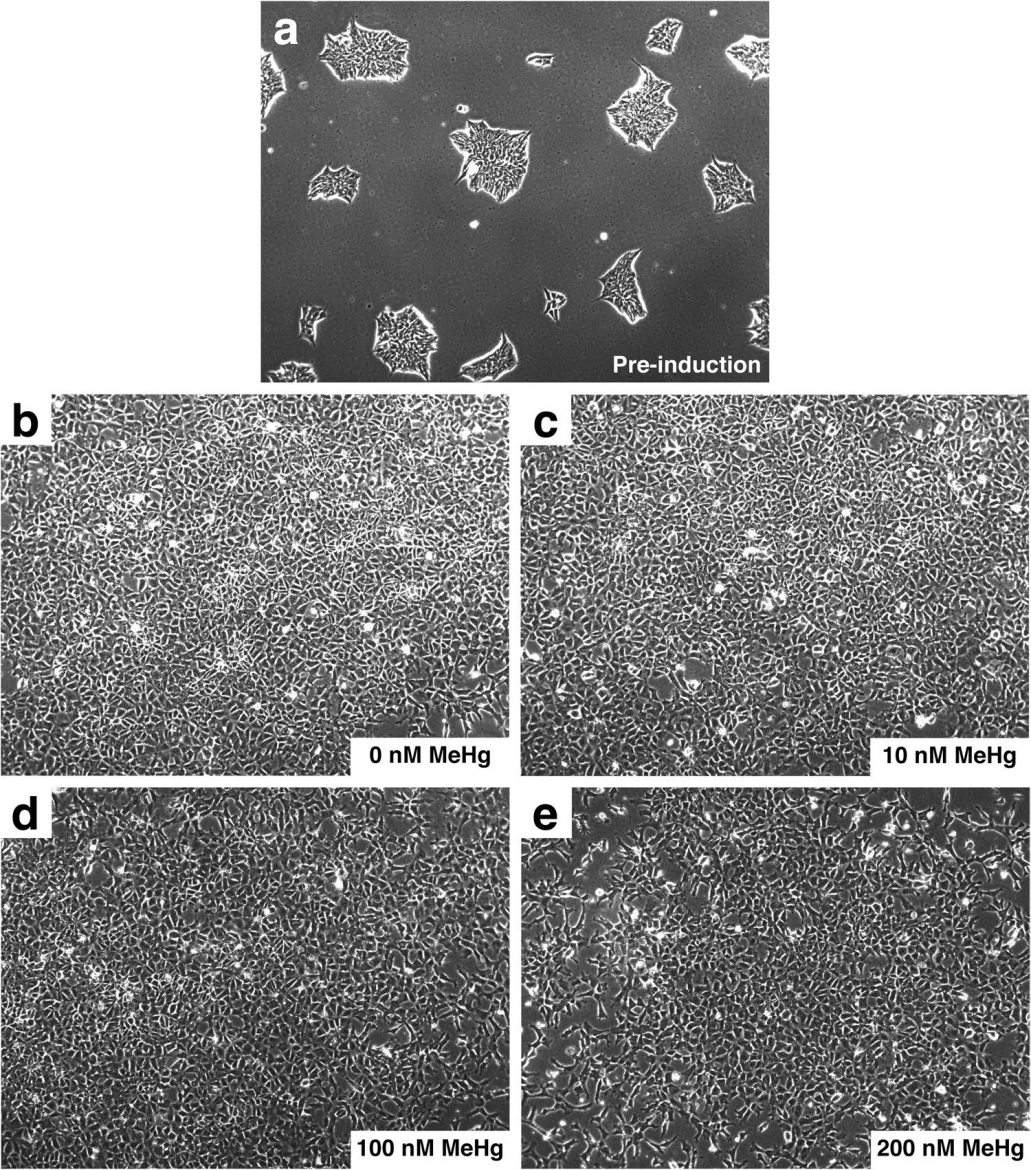
Cell attachment, colony formation, and morphology of definitive endoderm (DE) cells differentiated from human embryonic stem cells (hESCs) exposed to MeHg during DE differentiation. **a** Representative images of hESCs before induction. **b**–**e** Representative images of DE cells exposed to 0 nM, 10 nM, 100 nM, and 200 nM MeHg during DE cell differentiation. All images were taken under a 10× objective

To determine sub-lethal doses of MeHg exposure during hESC to DE differentiation, cell viability was measured following 0–10 μM of MeHg exposure. Differentiating cells exposed to 300 nM of MeHg showed a significant reduction in cell viability, and exposure to higher than 1 μM of MeHg resulted in almost complete cell death (Fig. 1). Exposure to concentrations at or below 200 nM of MeHg did not have a significant impact on cell viability, and differentiated DE cells showed no morphological difference when compared to the controls (Fig. 2). Therefore, the doses of 0, 10, 100, and 200 nM MeHg were identified as the sub-lethal doses for further transcriptomic analysis.

### Identification of differentially expressed genes (DEGs)

**Fig. 3:**
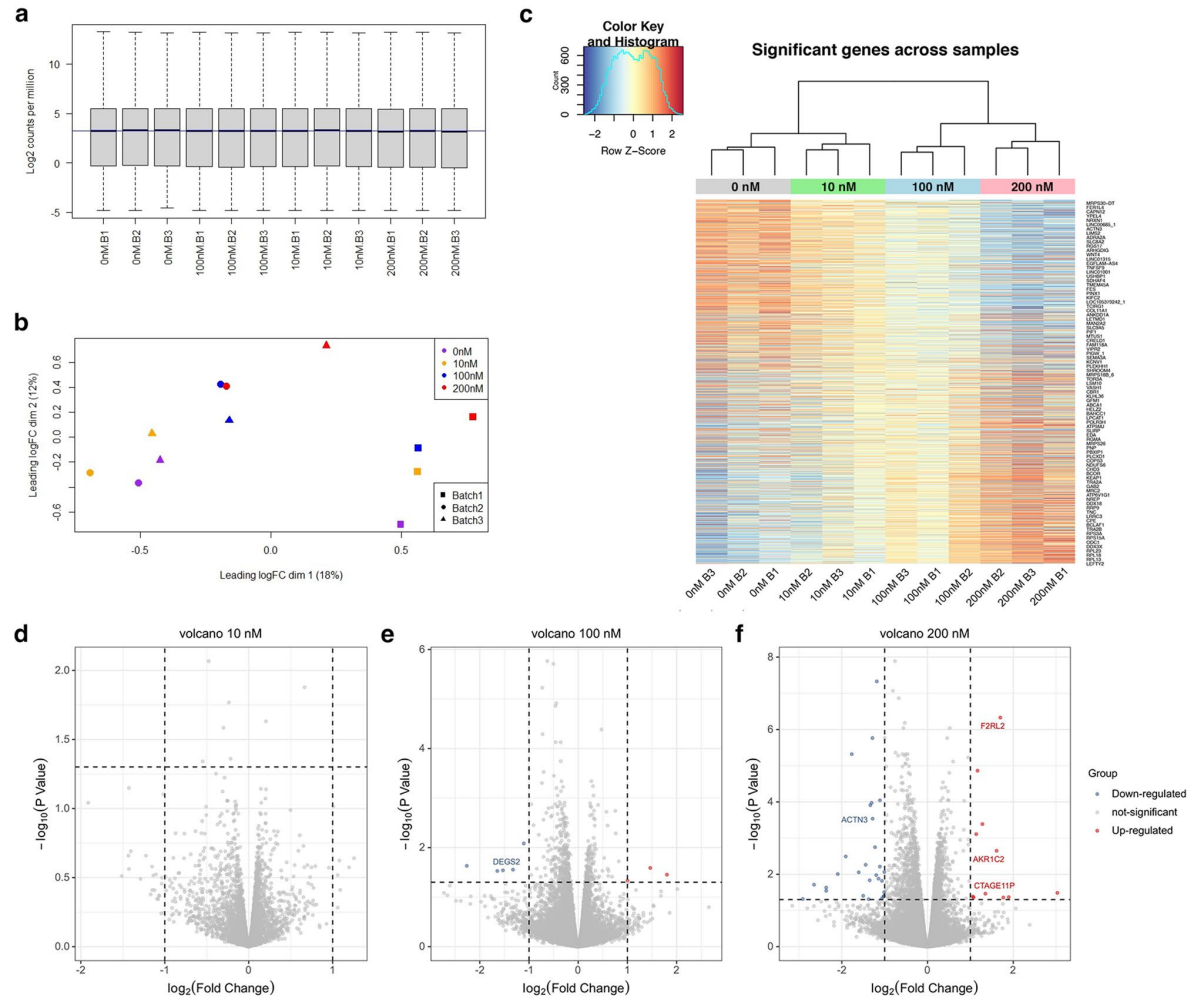
Data processing and identification of differentially expressed genes (DEGs). **a** Boxplot for transcriptomic samples after quantile normalization. **b** PCA for transcriptomic samples after quantile normalization. **c** The cluster heat map of DEGs with each row corresponding to a gene and each column corresponding to a sample. Light blue shows relatively low gene expression levels, whereas red represents relatively high gene expression levels. **d**–**f** Volcano plots of DEGs identified from 10 nM, 100 nM, and 200 nM MeHg exposure during human embryonic stem cell (hESC) to DE differentiation (color figure online)

Differentially expressed genes were identified with the “edgeR” package following data cleanup and quantile normalization of the samples (Fig. 3a, b). Based on the cutoff for DEGs (adjusted *p* value < 0.05 and absolute log2 FC > 1), a total of 1163 DEGs were identified (Excel Table S1), with 26 DEGs in the 10 nM treatment group, 393 DEGs in the 100 nM treatment group, and 1136 DEGs in the 200 nM treatment group. A total of 16 genes were differentially expressed in all 3 treatment groups. These DEGs were further visualized via a heat map (Fig. 3c) and a volcano plot (Fig. 3d–f). The heat map showed a clear clustering of each MeHg treatment group.

### Functional analysis of DEGs

**Fig. 4:**
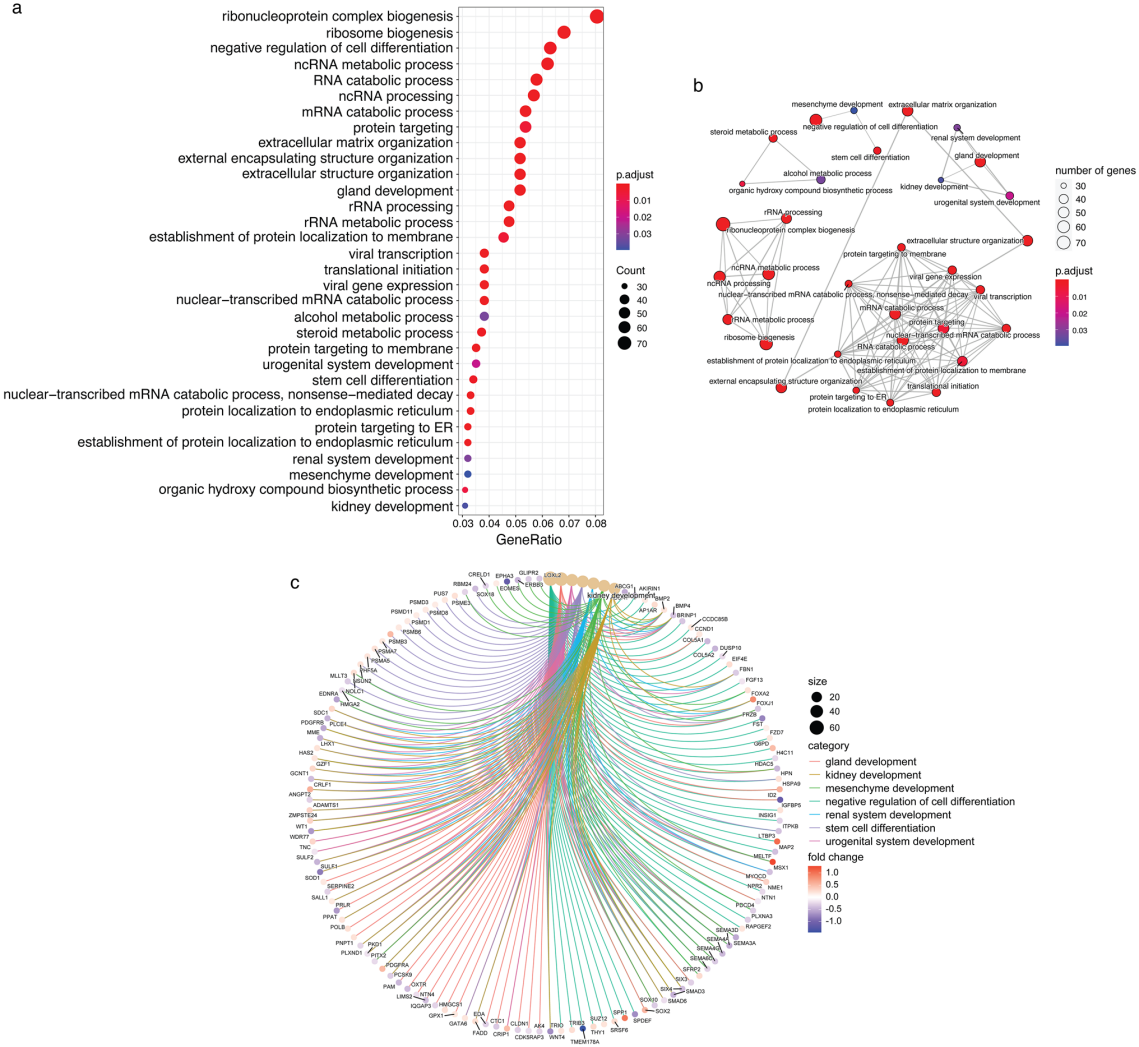
Significant enrichment GO terms of differentially expressed genes (DEGs). **a** GO functional enrichment dot plots of DEGs. The dots size represents the number of genes among the significant DEGs associated with the GO term. The color of the dots represents their significance in accordance with the adjusted *p* value. **b** Enrichment map of the 32 GO terms identified. **c** Gene-concept network plot of the seven GO terms related to early embryo development

The 1163 DEGs were analyzed for GO functional enrichment with a cutoff of *p* adj < 0.05. A total of 87 GO terms were identified to be significant, and among those, 32 GO terms with greater than 30 count number were kept for further analysis (Fig. 4a). The clustering of these 32 significant GO terms was organized into an enrichment map (Fig. 4b), showing that many of the significant GO terms were related to RNA metabolic processes including ribonucleoprotein complex biogenesis (GO:0022613), ribosome biogenesis (GO:0042254), ncRNA metabolic process (GO:0034660), ncRNA processing (GO:0034470), rRNA processing (GO:0006364), rRNA metabolic process (GO:0016070), and nuclear-transcribed mRNA catabolic process, nonsense-mediated decay (GO:0000184) or protein synthesis/transport such as mRNA catabolic process (GO:0006402), protein targeting (GO:0006605), establishment of protein localization to membrane (GO:0090150), translation initiation (GO:0006413), and protein targeting to membrane (GO:0006612). In addition, seven of these GO terms, including negative regulation of cell differentiation (GO:0045596), gland development (GO: 0048732), urogenital system development (GO: 0001655), stem cell differentiation (GO: 0048863), renal system development (GO:0072001), mesenchyme development (GO:0060485), and kidney development (GO:0001822) were GO terms that were linked to the GO term developmental process (GO:0032502), and the genes that are linked to these seven GO terms are shown in Fig. 4c. To select gene markers of interest for verification, a protein–protein interaction (PPI) network analysis of the genes within each of these seven development-related GO terms was conducted, and the top ten hub genes were identified from each of these seven GO terms. A total of 27 hub genes were identified after accounting for repetitions, with BMP4 being a hub gene in all 7 GO terms, and BMP2, WNT4, and SMAD3 appearing as hub genes in six out of the seven GO terms (Excel Table S2).

**Fig. 5:**
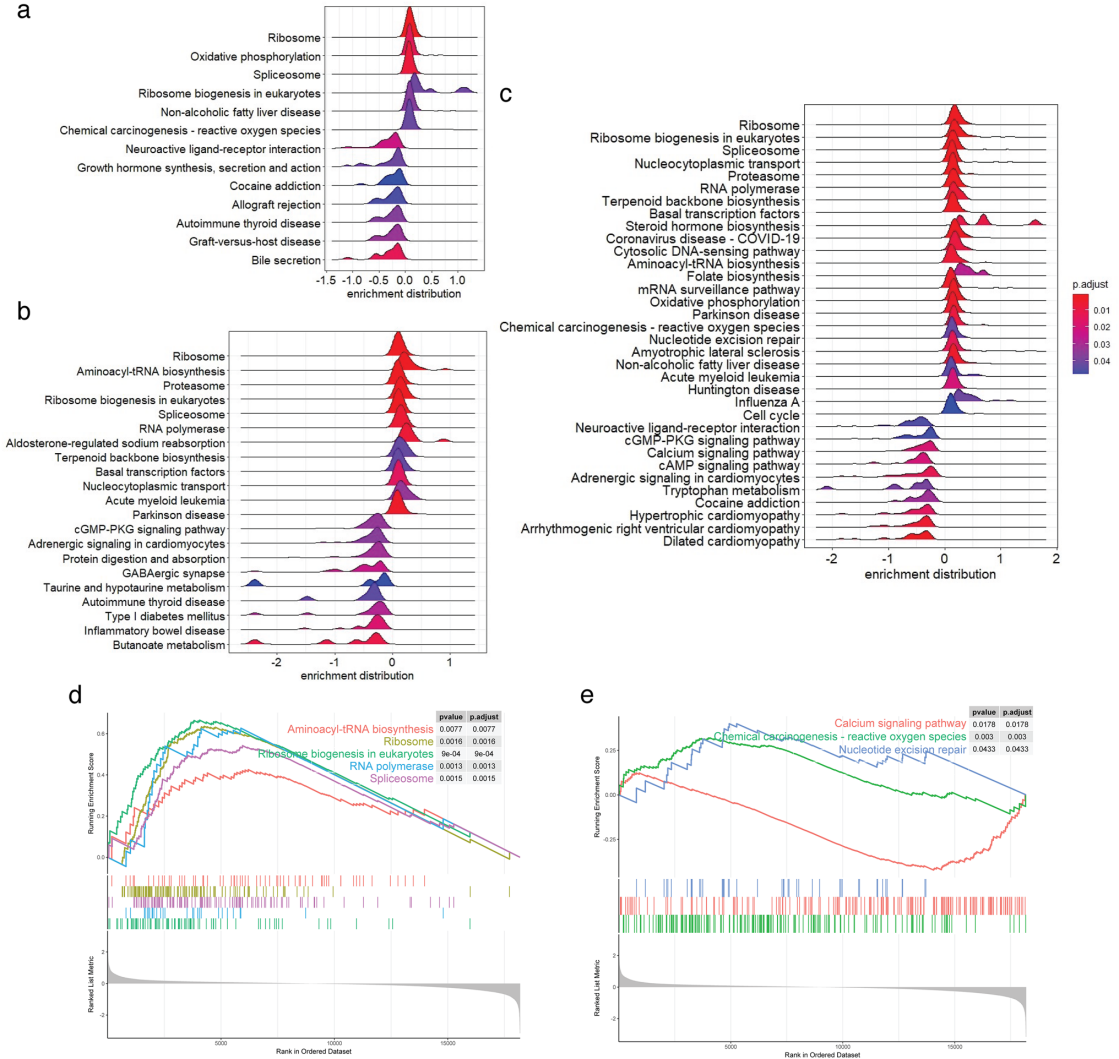
KEGG pathway enrichment of definitive endoderm (DE) cells exposed to **a** 10 nM, **b** 100 nM, and **c** 200 nM MeHg during human embryonic stem cell to DE cell differentiation. **d** Enrichment plot of ribosome and protein translation related KEGG pathways in 200 nM MeHg-dosed group compared to the controls. **e** Enrichment plot of KEGG pathways known to be perturbed by MeHg in 200 nM MeHg-dosed group compared to the controls

The KEGG pathway enrichment analysis (Fig. 5) also yielded similar results as the GO functional enrichment, with multiple ribosome-related pathways such as ribosome (hsa03010), ribosome biogenesis in eukaryotes (hsa03008), spliceosome (hsa03040), and RNA polymerase (hsa03020) being upregulated in all three MeHg treatment groups (Fig. 5a–c). In addition, pathways that are known to be perturbed by MeHg (Farina et al. 2011; Grotto et al. 2009; Novo et al. 2021; Roos et al. 2012), such as calcium signaling pathway (hsa04020), nucleotide excision repair (hsa03420), and chemical carcinogenesis–reactive oxygen species (hsa05208), are also reflected in the KEGG pathway enrichment analysis (Fig. 5e). In addition, according to the average rank of DEGs based on their BMDs (Excel Table S3), 21 KEGG pathways were successfully ranked with protein digestion and absorption (hsa04974) and calcium signaling pathway (hsa04020) considered as the early responses triggered by increasing doses of MeHg (Excel Table S4).

**Fig. 6:**
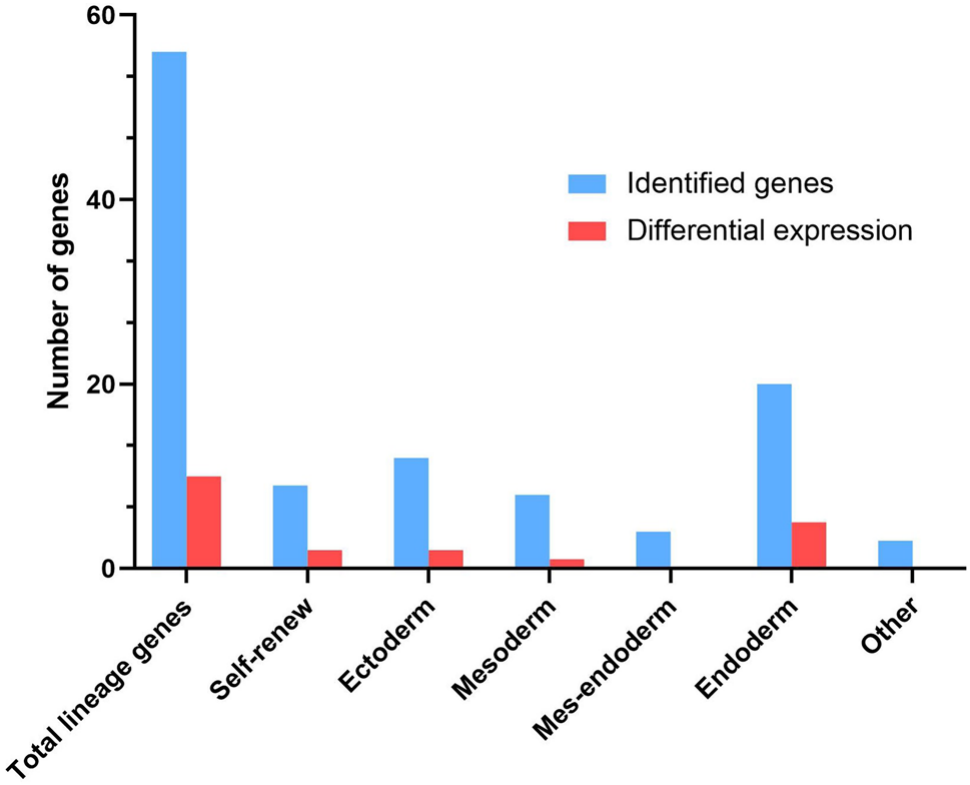
Lineage genes identified within the transcriptomics data set and the number of lineage genes that were differentially expressed following MeHg exposure during human embryonic stem cell to definitive endoderm differentiation

Lineage marker genes within DEGs were also identified based on previously published lineage genes (Bock et al. 2011; Tsankov et al. 2015), and ten lineage genes were found to be differentially expressed (Fig. 6). These included mesodermal/endodermal genes PDGFRA, FOXA2, GATA6, CLDN1, EOMES, and LEFTY2, ectodermal genes COL2A1 and MAP2, and self-renewal genes DNMT3B and SOX2. Of these genes, CLDN1, LEFTY2, and COL2A1 were downregulated following 200 nM of MeHg exposure during DE differentiation, whereas all the other lineage genes were upregulated.

As BMP4, SMAD3, FOXA2, GATA6, EOMES, and LEFTY2 were also major genes involved in the endoderm differentiation (Aksoy et al. 2014; Chu et al. 2016; Faial et al. 2015; Teo et al. 2011; Xuan and Sussel 2016), these genes were chosen for further downstream qPCR and western blot verification. In addition to the above genes, two other genes BHMT and URB1 were also selected for downstream verification. BHMT was chosen based on the justification that it has the biggest fold change among all DEGs, and BHMT itself is highly expressed in the endoderm-derived organs such as the liver and pancreas (Pajares and Pérez-Sala 2006), and URB1 was chosen as it is a ribosome biogenesis protein that can modulate endoderm-derived organ development (He et al. 2017), and ribosomal pathways were top enriched in both GO functional analysis and KEGG pathway analysis.

### RT-qPCR and western blot verification

**Fig. 7:**
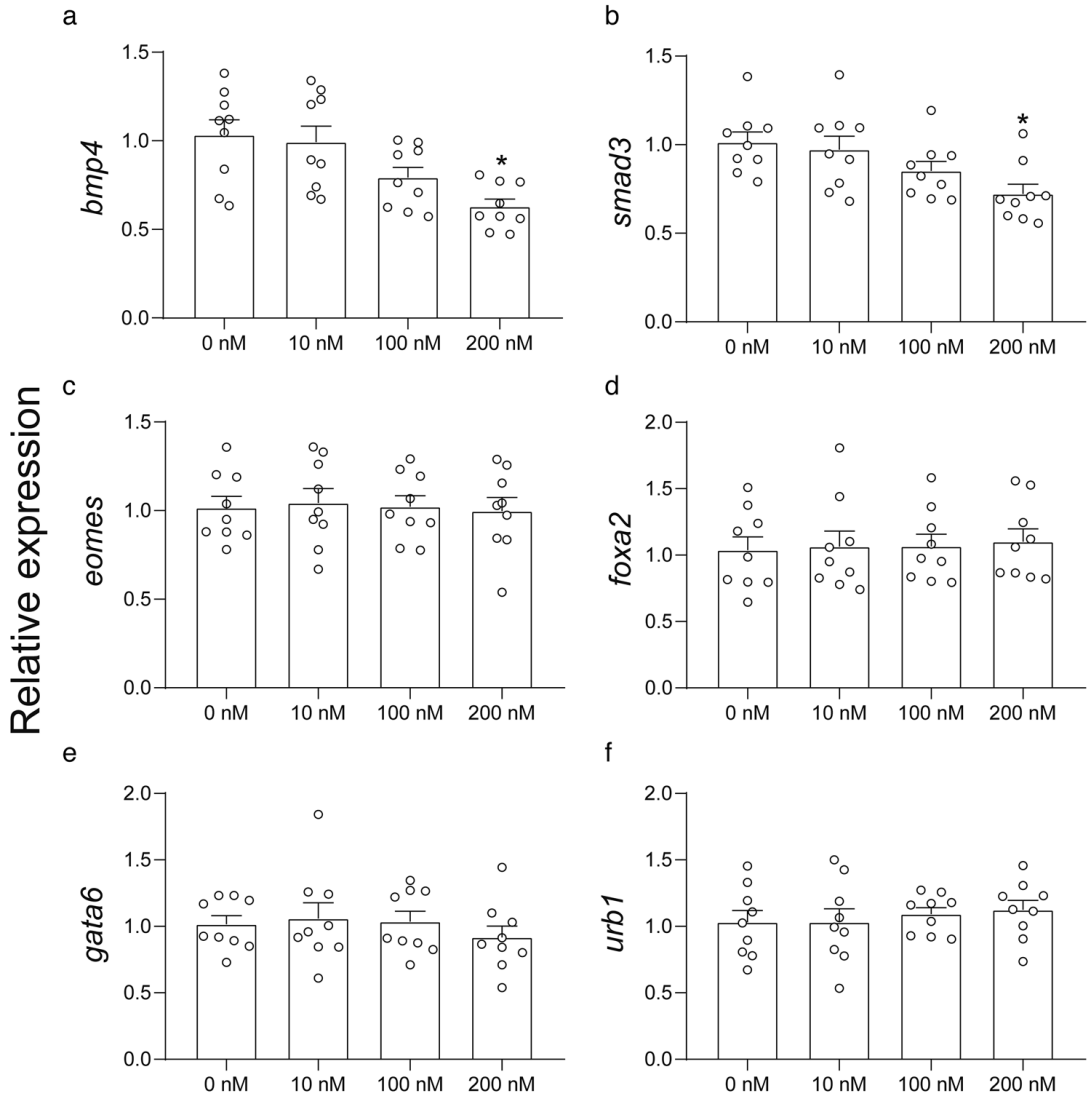
Expression of bmp4, smad3, eomes, foxa2, gata6, and urb1 genes in definitive endoderm (DE) cells exposed to MeHg during human embryonic stem cells to DE cell differentiation. Data are presented as means ± SEMs with *N* = 9 for each gene. The small circle symbols represent individual values of each experiment. One-way ANOVA and Dunnett’s post hoc test were used at each time point to compare differences between treatment and control groups with *p* < 0.05 being considered as significant marked by asterisk

Gene expression of the above-mentioned genes was further verified using RT-qPCR. Significant downregulation of *bmp4* (Fig. 7a) and *smad3* (Fig. 7b) was observed in the 200 nM MeHg treatment group, showing the same trends as the transcriptomics dataset. However, the other four genes [*eomes*, *foxa2*, *gata6*, *urb1* (Fig. 7c–f)] tested, which showed a significant but low fold change difference in the transcriptomics dataset, did not show any statistical differences when analyzed using RT-qPCR.

**Fig. 8:**
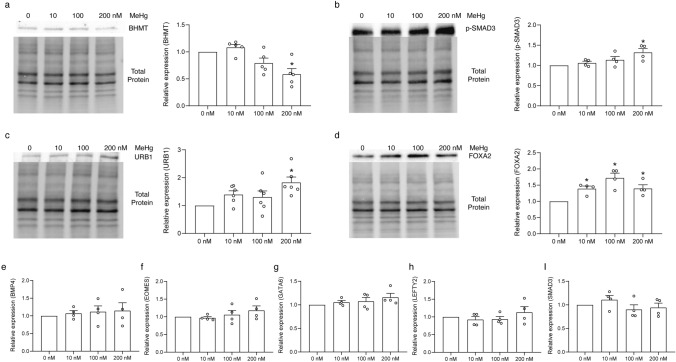
Expression of BHMT, p-SMAD3, URB1, FOXA2, BMP4, EOMES, GATA6, LEFTY2, and SMAD3 protein in definitive endoderm (DE) cells exposed to MeHg during human embryonic stem cells to DE cell differentiation. Data are presented as means ± SEMs with *N* = 4–6 for each protein. Each batch was first normalized to 0 nM exposure group before comparison. The small circle symbols represent individual values of each experiment. One-way ANOVA and Dunnett’s post hoc test were used at each time point to compare differences between treatment and control groups with *p* < 0.05 being considered as significant marked by asterisk

At the protein level, BHMT (Fig. 8a) showed a significant downregulation following 200 nM of MeHg exposure, phosphorylated SMAD3 (p-SMAD3) (Fig. 8b) and URB1 (Fig. 8c) showed a significant upregulation following 200 nM of MeHg exposure, whereas FOXA2 (Fig. 8d) was significantly upregulated at all three MeHg doses. BMP4, EOMES, GATA6, LEFTY2, and SMAD3 (Fig. 8e–i) did not show any significant differences following MeHg treatment, and neither did any of the autophagy markers [SQSTM1, LC3BII/LC3BI (Fig. S2)].

## Discussion

The main goal of the present study was to assess the influence of MeHg exposure on the development of human embryonic stem cells (hESCs) during definitive endoderm (DE) cell differentiation using a transcriptomics approach. Our results showed that MeHg targeted mainly ribosomal processes to alter protein synthesis and translocation during hESCs to DE cell differentiation, resulting in an overall enhancement of cell differentiation.

The viability of differentiating cells was not affected when exposed to MeHg doses below or at 200 nM, whereas MeHg doses above 200 nM decreased its viability in a dose-dependent manner. In contrast, undifferentiated hESCs showed close to 50% reduction in cell viability when exposed to 200 nM of MeHg for the same duration (Li et al. 2021), suggesting that the hESCs which are differentiating toward DE cells have a higher tolerance for MeHg exposure in terms of cell viability when compared to undifferentiated hESCs. However, this does not indicate that MeHg exposure under 200 nM has no effect on these differentiating hESCs, as sub-lethal doses of MeHg exposure have been shown to alter protein expression and induce cognitive deficits in animal models (Bittencourt et al. 2019). This is supported by findings in this study with respect to the effects of MeHg at sub-lethal doses on the altered gene and protein expressions of hESCs during the differentiation toward DE cells.

The transcriptomics dataset showed that MeHg exposure at 10 nM affected the least number of genes, with only 26 genes showing a significant difference in expression pattern compared to the vehicle control. The number of differentially expressed genes (DEGs) increased as MeHg concentrations increased, but the genes that were affected remained mostly consistent, as 85% of DEGs in the 10 nM MeHg-dosed group and 93% of DEGs in the 100 nM MeHg-dosed group were also DEGs in the 200 nM MeHg-dosed group, suggesting a generally similar manner in gene pattern alterations among the three doses. Functional analysis of DEGs via GO functional enrichment or KEGG pathway analysis also confirmed that MeHg disrupted hESCs differentiation into DE cells via similar pathways, with ribosomal and protein synthesis/translocation pathways consistently appearing in all three MeHg-dosed groups. Thus, it is likely that MeHg exerts its developmental toxicity mainly by disrupting ribosome biogenesis during early cell lineage differentiation.

It is well-recognized that ribosome biogenesis is important for the maintenance of stem cell pluripotency and its subsequent differentiation (Gabut et al. 2020; Saba et al. 2021; Tahmasebi et al. 2019). In undifferentiated stem cells, ribosome biogenesis is generally at a high level, though global protein synthesis rates tend to be low, which primes the undifferentiated stem cell for rapid and efficient reassembly of their proteome into the target cell types during differentiation. During differentiation, rates of protein synthesis and ribosome biogenesis are tightly and dynamically regulated in accordance with the needs of the differentiating cells, with protein synthesis rates increasing during differentiation. In contrast, ribosome biogenesis transiently decreases only during the early phases of differentiation and then rises back up to high levels (Baser et al. 2019; Corsini et al. 2018). Following MeHg exposure, upregulation of genes within ribosomal-related processes enriched from GO terms, upregulation of the ribosome biogenesis homolog URB1, and the upregulation of the KEGG pathway ribosome biogenesis in eukaryotes (hsa03008) all suggest that MeHg upregulates ribosomal biogenesis during hESC to DE cell differentiation. Despite undifferentiated stem cells having high ribosome biogenesis, and MeHg exposure on undifferentiated hESCs showing enhanced pluripotency (Li et al. 2021), it is not likely that MeHg exposure during hESC to DE cell differentiation inhibited differentiation and enhanced hESCs’ pluripotency. Firstl, despite “negative regulation of cell differentiation” being enriched through GO functional enrichment, most genes within the GO term were downregulated following MeHg exposure (39 downregulated vs 27 upregulated), suggesting inhibition of negative regulation, and thus enhancement of cell differentiation. Second, multiple pieces of evidence within the dataset also point to an upregulation of protein synthesis, translocation, and turnover. This includes genes within the GO terms “translational inhibition” (seven upregulated and none downregulated) and “protein targeting” (nine upregulated and six downregulated) being mostly upregulated, and core enriched genes within the KEGG pathways for “spliceosome”, “RNA polymerase”, and “aminoacyl-tRNA biosynthesis” being upregulated. In addition, MeHg is capable of inducing protein synthesis in animal models, with increases in levels of liver ribosomes, ribosomal subunits, and polyribosomes, along with a threefold increase in the incorporation of labeled amino acids in the livers of rats exposed to MeHg (Brubaker et al. 1971). Thus, the combination of ribosome biosynthesis and protein synthesis upregulation suggests that MeHg upregulates ribosome biogenesis leading to increased global protein translation and enhancing differentiation which could further lead to the overgrowth of specific organs. For example, in zebrafish, MeHg caused upregulation of URB1 during DE differentiation, leading to alterations in ribosome biogenesis and overgrowth of digestive organs (He et al. 2017).

What is seemingly contradictory to this conclusion is that nine proteasome subunits (PSMA5, PSMA7, PSMB3, PSMB6, PSMD1, PSMD3, PSMD8, PSMD11, PSME3) and the KEGG pathway proteasome (hsa03050) were also all upregulated following MeHg exposure during hESC to DE cell differentiation. High levels of proteasome activity have been associated with the maintenance of pluripotency (Saez et al. 2018; Schröter and Adjaye 2014; Yan et al. 2020). However, studies have also shown that the ubiquitin proteasome pathway plays a role in modulating the toxicity of MeHg. Specifically, overexpression of the ubiquitin-protein ligase CDC34 significantly increased cellular-ubiquitinated protein levels in yeast and human cell lines, increasing their resistance to MeHg exposure (Hwang 2007; Hwang et al. 2002). Thus, the upregulation of the proteasome pathway may be due to cells resisting MeHg toxicity and not maintaining pluripotency.

Our results in the ranked KEGG pathways based on BMDs of the gene set involved showed that the calcium signaling pathway (hsa04020) is the most sensitive responses to MeHg exposure. This indicates that MeHg toxicity began with the disruption of calcium homeostasis which can result in the generation of excessive amounts of ROS (Farina and Aschner 2017). This is nicely supported in our data with KEGG pathways chemical carcinogenesis-reactive oxygen species (hsa05208) and oxidative phosphorylation (hsa00190) ranking behind the KEGG pathway calcium signaling pathway. Proteasome (hsa03050), which ranked behind chemical carcinogenesis-reactive oxygen species and oxidative phosphorylation, would then need to be upregulated to clean up the damaged proteins caused by the elevated oxidative stress. Meanwhile, the overproduction of ROS has also been linked to hyperactive ribosome biogenesis, and protein synthesis in animal embryos and human cell lines (Huang et al. 2021; Oliveira et al. 2019), and is considered to promote stem cell differentiation (Bigarella et al. 2014), including toward the DE fate (Lv et al. 2022).

Under normal circumstances, antioxidants such as glutathione can prevent oxidative stress through the removal of ROS. However, glutathione is also involved in Hg detoxification by specifically binding with MeHg to form a complex that prevents Hg from binding to cellular proteins and causing cellular damage (Kromidas et al. 1990) or by forming a glutathione-mercury complex in the liver for the excretion of mercury (Zalups 2000). Therefore, depleting existing glutathione concentrations will result in a decreased capacity for the removal of ROS and subsequent cell death. To respond to the depletion of glutathione by Hg, upregulation of the trans-sulfuration pathway that synthesizes glutathione from homocysteine (Hcy) has been observed (Woods and Ellis 1995). From our results, this upregulation of glutathione synthesis is reflected as a downregulation of betaine-homocysteine S-methyltransferase (BHMT), one of the genes with the biggest fold change following MeHg exposure. BHMT normally plays an important role in the regulation of Hcy metabolism by catalyzing the resynthesis of methionine from Hcy (Pajares and Pérez-Sala 2006). Thus, the downregulation of BHMT would facilitate Hcy in entering the trans-sulfuration pathway for glutathione synthesis. Interestingly, folate biosynthesis (hsa00790), which produces folate, that also catalyzes the resynthesis of methionine from Hcy was significantly upregulated following MeHg exposure. This could partly reflect the lack of methionine within the cell due to Hcy being directed for glutathione synthesis to protect against oxidative stress induced by MeHg exposure.

**Fig. 9:**
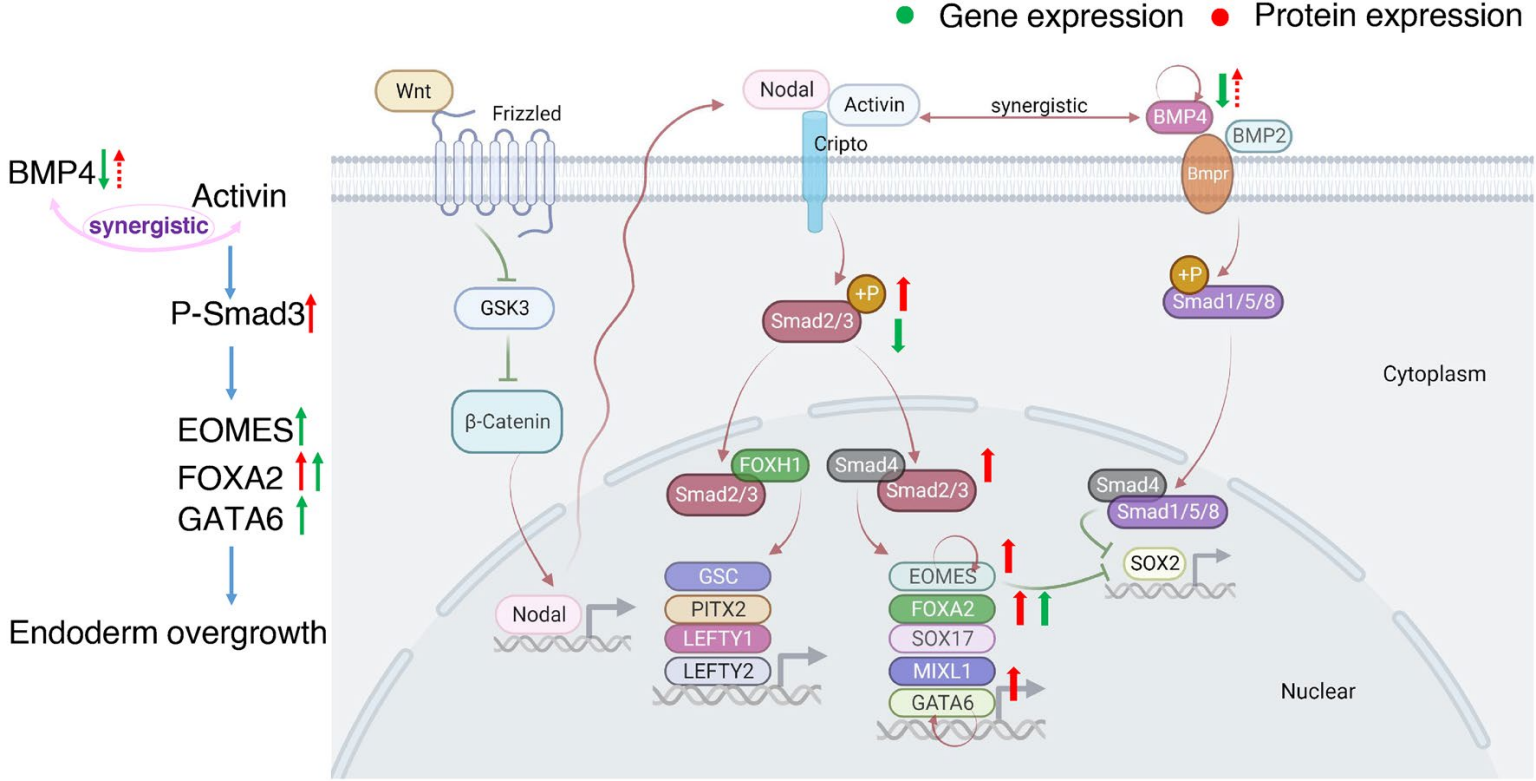
Summary of genes/proteins involved in definitive endoderm differentiation and changes in these genes/proteins following MeHg exposure during differentiation. See text for more information

As MeHg resulted in enhanced differentiation during hESC to DE cell differentiation, it is likely that endodermal fate was over-promoted. Under normal conditions, three major extracellular signals, Wnt, Nodal/Activin, and BMPs, regulate definitive endoderm differentiation. These signals all, in turn, phosphorylate SMAD2/3 proteins (Faial et al. 2015; Funa et al. 2015; Teo et al. 2011), which depending on the transcriptional regulator, it binds to either activate endodermal fate genes when bound to SMAD4 (Yang and Jiang 2020) or activate mesodermal fate genes when bound to FOXH1 (Charney et al. 2017). In addition, BMP4 can act synergistically with activin to further upregulate the expression of transcription factors involved in endoderm differentiation and promote the endodermal fate (Teo et al. 2012). BMP4 itself can also cooperate with FGF2 via ERK to further induce mesoderm and inhibit the endoderm differentiation (Bernardo et al. 2011). To understand how MeHg may have disrupted stem cell specification during the differentiation toward DE cells, we examined lineage gene markers within our dataset. We found that the most significantly affected genes by MeHg were mesodermal or endodermal genes, including *pdgfra*, *foxa2*, *gata6*, *cldn1*, *eomes* and *lefty2*. Most notably, the expression of endodermal genes *foxa2*, *eomes*, and *gata6* were upregulated, and FOXA2 was confirmed to be upregulated at the protein level as well. FOXA2 is an important transcription factor that is expressed during early endoderm differentiation and, when overexpressed, can lead to the expression of genes associated with endodermal lineage, but does not induce the expression of genes involved in late endoderm differentiation (Levinson-Dushnik and Benvenisty 1997). EOMES, on the other hand, interacts with SMAD2/3 to initiate the transcriptional network governing endoderm formation and, when overexpressed, leads to the expression of DE markers such as FOXA2 (Faial et al. 2015; Teo et al. 2011). These changes are likely initiated by the upregulation of phosphorylated SMAD3 (p-SMAD3) though *smad3* gene expression was significantly downregulated in this study, suggestive of a negative feedback loop between p-SMAD3 and *smad3* gene expression. Like *smad3* gene and p-SMAD3 protein, there might be a negative feedback loop for BMP4 regulation as well, as bmp4 gene was downregulated, yet BMP4 protein showed an increasing though non-significant trend following MeHg exposure, which could further promote endoderm differentiation. Thus, MeHg exposure during hESC to DE cell differentiation leads to overexpression of endodermal genes, which may ultimately result in the over-promotion of endodermal fate (Fig. 9).

**Fig. 10:**
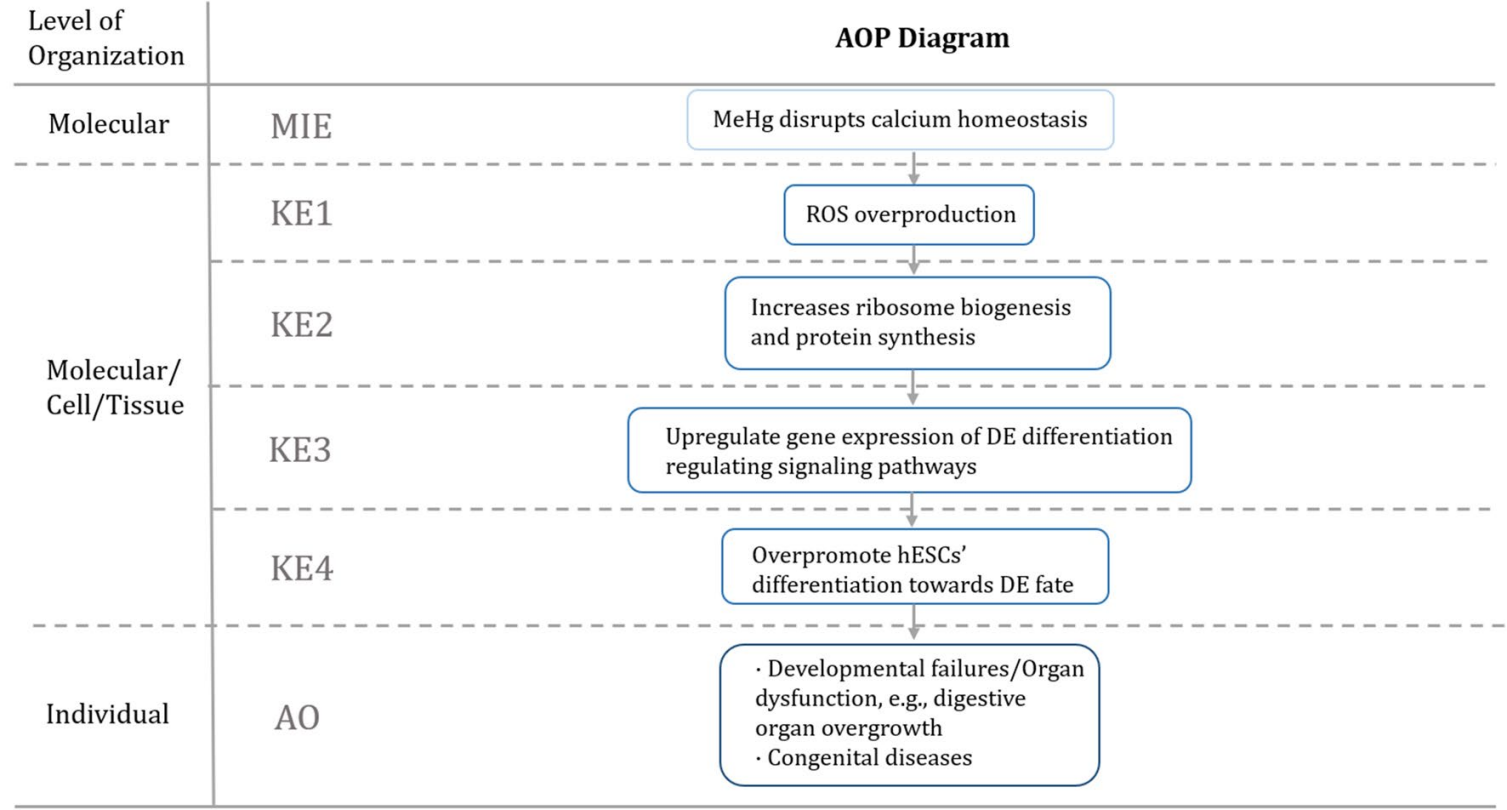
Adverse outcome pathway diagram of the effects of MeHg exposure during human embryonic stem cell to definitive endoderm differentiation

In summary, our study showed that MeHg exposure at environmentally relevant doses during hESC to DE differentiation has sub-lethal effects that could cause global gene expression changes, leading to calcium dyshomeostasis and ROS overproduction, resulting in an upregulation of ribosome biogenesis and global protein synthesis/translocation to promote hESC differentiation. Considering that the key transcription factors such as EOMES and FOXA2, which are critical in endodermal development, are overexpressed following MeHg exposure, hESC specification could be affected with endodermal fate being over-promoted, abnormal organ development, and subsequently congenital diseases later in life. A summary of this effect is presented in the form of an adverse outcome pathway in Fig. 10. It is not possible to extrapolate the dose–response relationship observed in this in vitro study to humans. However, the treatment doses covered a range of total Hg typically found in human maternal blood (0.1–284.7 nM) (Table 1). Our results suggest that the risk of MeHg during pregnancy, especially the newfound effects on endodermal development, needs more consideration for public health promotion and disease prevention.

## Supplementary Information

Below is the link to the electronic supplementary material.

Supplementary file1 (XLSX 165 KB)

Supplementary file2 (XLSX 10 KB)

Supplementary file3 (XLSX 54 KB)

Supplementary file4 (XLSX 9 KB)

Supplementary file5 (DOCX 1424 KB)

## Data Availability

Data will be available upon request.

## References

[CR1] Aksoy I, Giudice V, Delahaye E (2014). Klf4 and Klf5 differentially inhibit mesoderm and endoderm differentiation in embryonic stem cells. Nat Commun.

[CR2] Amin-Zaki L, Majeed M, Greenwood MR, Elhassani SB, Clarkson TW, Doherty RA (1981). Methylmercury poisoning in the Iraqi suckling infant: a longitudinal study over five years. J Appl Toxicol.

[CR3] Bakir F, Damluji SF, Amin-Zaki L (1973). Methylmercury poisoning in Iraq: an interuniversity report. Science.

[CR4] Baser A, Skabkin M, Kleber S (2019). Onset of differentiation is post-transcriptionally controlled in adult neural stem cells. Nature.

[CR5] Bernardo AS, Faial T, Gardner L (2011). BRACHYURY and CDX2 mediate BMP-induced differentiation of human and mouse pluripotent stem cells into embryonic and extraembryonic lineages. Cell Stem Cell.

[CR6] Bigarella CL, Liang R, Ghaffari S (2014). Stem cells and the impact of ROS signaling. Development.

[CR7] Bittencourt LO, Dionizio A, Nascimento PC (2019). Proteomic approach underlying the hippocampal neurodegeneration caused by low doses of methylmercury after long-term exposure in adult rats. Metallomics.

[CR8] Bock C, Kiskinis E, Verstappen G (2011). Reference Maps of human ES and iPS cell variation enable high-throughput characterization of pluripotent cell lines. Cell.

[CR9] Bose R, Onishchenko N, Edoff K, Janson Lang AM, Ceccatelli S (2012). Inherited effects of low-dose exposure to methylmercury in neural stem cells. Toxicol Sci.

[CR10] Brubaker P, Lucier G, Klein R (1971). The effects of methylmercury on protein synthesis in rat liver. Biochem Biophys Res Commun.

[CR88] Caetano T, Branco V, Cavaco A, Carvalho C (2019). Risk assessment of methylmercury in pregnant women and newborns in the island of Madeira (Portugal) using exposure biomarkers and food-frequency questionnaires. J Toxicol Environ Health A.

[CR11] Carneiro MFH, Souza JMO, Grotto D, Batista BL, de Oliveira Souza VC, Barbosa F (2014). A systematic study of the disposition and metabolism of mercury species in mice after exposure to low levels of thimerosal (ethylmercury). Environ Res.

[CR12] Castoldi AF, Onishchenko N, Johansson C (2008). Neurodevelopmental toxicity of methylmercury: laboratory animal data and their contribution to human risk assessment. Regul Toxicol Pharmacol.

[CR13] Chang J-W, Chen H-L, Su H-J, Liao P-C, Guo H-R, Lee C-C (2011). Simultaneous exposure of non-diabetics to high levels of dioxins and mercury increases their risk of insulin resistance. J Hazard Mater.

[CR14] Charney RM, Forouzmand E, Cho JS (2017). Foxh1 occupies cis-regulatory modules prior to dynamic transcription factor interactions controlling the mesendoderm gene program. Dev Cell.

[CR15] Chen YW, Huang CF, Tsai KS (2006). Methylmercury induces pancreatic β-cell apoptosis and dysfunction. Chem Res Toxicol.

[CR16] Chu L-F, Leng N, Zhang J (2016). Single-cell RNA-seq reveals novel regulators of human embryonic stem cell differentiation to definitive endoderm. Genome Biol.

[CR17] Clarkson TW (2002). The three modern faces of mercury. Environ Health Perspect.

[CR18] Corsini NS, Peer AM, Moeseneder P (2018). Coordinated control of mRNA and rRNA processing controls embryonic stem cell pluripotency and differentiation. Cell Stem Cell.

[CR19] Cuello S, Goya L, Madrid Y (2010). Molecular mechanisms of methylmercury-induced cell death in human HepG2 cells. Food Chem Toxicol.

[CR20] da Rosa-Silva HT, Panzenhagen AC, Schmidtt V (2020). Hepatic and neurobiological effects of foetal and breastfeeding and adulthood exposure to methylmercury in Wistar rats. Chemosphere.

[CR89] Donohue A, Wagner CL, Burch JB, Rothenberg SE (2018). Blood total mercury and methylmercury among pregnant mothers in Charleston, South Carolina, USA. J Expo Sci Environ Epidemiol.

[CR21] dos Santos AA, Hort MA, Culbreth M (2016). Methylmercury and brain development: a review of recent literature. J Trace Elem Med Biol.

[CR22] Driscoll CT, Mason RP, Chan HM, Jacob DJ, Pirrone N (2013). Mercury as a global pollutant: sources, pathways, and effects. Environ Sci Technol.

[CR23] Eto K (1997). Pathology of minamata disease. Toxicol Pathol.

[CR24] Eto K, Marumoto M, Takeya M (2010). The pathology of methylmercury poisoning (minamata disease) the 50th anniversary of Japanese society of neuropathology. Neuropathology.

[CR25] Ewald J, Soufan O, Xia J, Basu N (2021). FastBMD: an online tool for rapid benchmark dose–response analysis of transcriptomics data. Bioinformatics.

[CR26] Faial T, Bernardo AS, Mendjan S (2015). Brachyury and SMAD signalling collaboratively orchestrate distinct mesoderm and endoderm gene regulatory networks in differentiating human embryonic stem cells. Development.

[CR27] Farina M, Aschner M (2017). Methylmercury-induced neurotoxicity: focus on pro-oxidative events and related consequences. Adv Neurobiol.

[CR28] Farina M, Rocha JB, Aschner M (2011). Mechanisms of methylmercury-induced neurotoxicity: evidence from experimental studies. Life Sci.

[CR29] Feng L, Li P, Feng X (2021). Methylmercury bioaccumulation in rice and health effects: a systematic review. Curr Opin Environ Sci Health.

[CR30] Fujimura M, Usuki F (2015). Low concentrations of methylmercury inhibit neural progenitor cell proliferation associated with up-regulation of glycogen synthase kinase 3β and subsequent degradation of cyclin E in rats. Toxicol Appl Pharmacol.

[CR31] Funa NS, Schachter KA, Lerdrup M (2015). β-Catenin regulates primitive streak induction through collaborative interactions with SMAD2/SMAD3 and OCT4. Cell Stem Cell.

[CR90] Furgal C, Lemire M, Ayotte P et al (2018) Exposure to food chain contaminants in Nunavik: evaluating spatial and time trends among pregnant women & implementing effective health communication for healthy pregnancies and children (Year 2 of 3)

[CR32] Gabut M, Bourdelais F, Durand S (2020). Ribosome and translational control in stem cells. Cells.

[CR33] Gonzalez P, Dominique Y, Massabuau J, Boudou A, Bourdineaud J (2005). Comparative effects of dietary methylmercury on gene expression in liver, skeletal muscle, and brain of the zebrafish (Danio rerio). Environ Sci Technol.

[CR34] Grotto D, Barcelos GR, Valentini J (2009). Low levels of methylmercury induce DNA damage in rats: protective effects of selenium. Arch Toxicol.

[CR35] Harada M (1978). Congenital minamata disease: intrauterine methylmercury poisoning. Teratology.

[CR36] Harada M (1995). Minamata disease: methylmercury poisoning in Japan caused by environmental pollution. Crit Rev Toxicol.

[CR37] He X, Imanishi S, Sone H (2012). Effects of methylmercury exposure on neuronal differentiation of mouse and human embryonic stem cells. Toxicol Lett.

[CR38] He K, Xun P, Liu K, Morris S, Reis J, Guallar E (2013). Mercury exposure in young adulthood and incidence of diabetes later in life: the CARDIA trace element study. Diabetes Care.

[CR39] He J, Yang Y, Zhang J (2017). Ribosome biogenesis protein Urb1 acts downstream of mTOR complex 1 to modulate digestive organ development in zebrafish. J Genet Genom.

[CR40] Huang Y, Li Z, Lin E, He P, Ru G (2021). Oxidative damage-induced hyperactive ribosome biogenesis participates in tumorigenesis of offspring by cross-interacting with the Wnt and TGF-β1 pathways in IVF embryos. Exp Mol Med.

[CR41] Hwang G-W (2007). Ubiquitin-proteasome system as a factor that determine the sensitivity to methylmercury. Yakugaku Zasshi: J Pharma Soc Japan.

[CR42] Hwang GW, Furuchi T, Naganuma A (2002). Ubiquitin-proteasome system is responsible for the protection of yeast and human cells against methylmercury. FASEB J.

[CR91] Iwai-Shimada M, Kameo S, Nakai K (2019). Exposure profile of mercury, lead, cadmium, arsenic, antimony, copper, selenium and zinc in maternal blood, cord blood and placenta: the Tohoku Study of Child Development in Japan. Environ Health Prev Med.

[CR43] Jamalpoor A, Hartvelt S, Dimopoulou M (2022). A novel human stem cell-based biomarker assay for in vitro assessment of developmental toxicity. Birth Defect Res.

[CR92] Kobayashi S, Kishi R, Saijo Y (2019). Association of blood mercury levels during pregnancy with infant birth size by blood selenium levels in the Japan Environment and Children’s Study: a prospective birth cohort. Environ Int.

[CR44] Kromidas L, Trombetta LD, Jamall IS (1990). The protective effects of glutathione against methylmercury cytotoxicity. Toxicol Lett.

[CR45] Levinson-Dushnik M, Benvenisty N (1997). Involvement of hepatocyte nuclear factor 3 in endoderm differentiation of embryonic stem cells. Mol Cell Biol.

[CR46] Li B, Qiao C, Jin X, Chan HM (2021). Characterizing the low-dose effects of methylmercury on the early stages of embryo development using cultured human embryonic stem cells. Environ Health Perspect.

[CR47] Liao Y, Smyth GK, Shi W (2019). The R package Rsubread is easier, faster, cheaper and better for alignment and quantification of RNA sequencing reads. Nucleic Acids Res.

[CR93] Lukina AO, Fisher M, Khoury C (2021). Temporal variation of total mercury levels in the hair of pregnant women from the Maternal-Infant Research on Environmental Chemicals (MIREC) study. Chemosphere.

[CR48] Lv J, Yi Y, Qi Y (2022). Mitochondrial homeostasis regulates definitive endoderm differentiation of human pluripotent stem cells. Cell Death Discovery.

[CR49] Mela M, Randi M, Ventura D, Carvalho C, Pelletier E, Ribeiro CO (2007). Effects of dietary methylmercury on liver and kidney histology in the neotropical fish Hoplias malabaricus. Ecotoxicol Environ Saf.

[CR50] Muhr J, Ackerman KM (2020) Embryology, gastrulation. PMID: 32119281 32119281

[CR51] Nagashima K (1997). A review of experimental methylmercury toxicity in rats: neuropathology and evidence for apoptosis. Toxicol Pathol.

[CR52] Novo JP, Martins B, Raposo RS (2021). Cellular and molecular mechanisms mediating methylmercury neurotoxicity and neuroinflammation. Int J Mol Sci.

[CR53] Oliveira V, Mahajan N, Bates ML (2019). The snoRNA target of t (4; 14) in multiple myeloma regulates ribosome biogenesis. FASEB BioAdv.

[CR54] Pajares MA, Pérez-Sala D (2006). Betaine homocysteine S-methyltransferase: just a regulator of homocysteine metabolism?. Cell Mol Life Sci CMLS.

[CR55] Parks JM, Johs A, Podar M (2013). The genetic basis for bacterial mercury methylation. Science.

[CR56] Prince LM, Neely MD, Warren EB (2021). Environmentally relevant developmental methylmercury exposures alter neuronal differentiation in a human-induced pluripotent stem cell model. Food Chem Toxicol.

[CR57] Rao X, Huang X, Zhou Z, Lin X (2013). An improvement of the 2ˆ (–delta delta CT) method for quantitative real-time polymerase chain reaction data analysis. Biostat Bioinform Biomath.

[CR58] Robinson MD, McCarthy DJ, Smyth GK (2010). edgeR: a Bioconductor package for differential expression analysis of digital gene expression data. Bioinformatics.

[CR59] Roos D, Seeger R, Puntel R, Vargas Barbosa N (2012). Role of calcium and mitochondria in MeHg-mediated cytotoxicity. J Biomed Biotechnol.

[CR94] Rothenberg SE, Yu X, Zhang Y (2013). Prenatal methylmercury exposure through maternal rice ingestion: insights from a feasibility pilot in Guizhou Province, China. Environ Pollut.

[CR60] Saba JA, Liakath-Ali K, Green R, Watt FM (2021). Translational control of stem cell function. Nat Rev Mol Cell Biol.

[CR61] Saez I, Koyuncu S, Gutierrez-Garcia R, Dieterich C, Vilchez D (2018). Insights into the ubiquitin-proteasome system of human embryonic stem cells. Sci Rep.

[CR62] Schröter F, Adjaye J (2014). The proteasome complex and the maintenance of pluripotency: sustain the fate by mopping up?. Stem Cell Res Ther.

[CR63] Schumacher L, Abbott LC (2017). Effects of methyl mercury exposure on pancreatic beta cell development and function. J Appl Toxicol.

[CR64] Seiler AE, Spielmann H (2011). The validated embryonic stem cell test to predict embryotoxicity in vitro. Nat Protoc.

[CR65] Sharma K, Asp NT, Harrison S (2022). Autophagy modulates cell fate decisions during lineage commitment. Autophagy.

[CR66] Streets DG, Zhang Q, Wu Y (2009). Projections of global mercury emissions in 2050. Environ Sci Technol.

[CR67] Stummann T, Hareng L, Bremer S (2007). Embryotoxicity hazard assessment of methylmercury and chromium using embryonic stem cells. Toxicology.

[CR68] Stummann TC, Hareng L, Bremer S (2009). Hazard assessment of methylmercury toxicity to neuronal induction in embryogenesis using human embryonic stem cells. Toxicology.

[CR69] Tahmasebi S, Amiri M, Sonenberg N (2019). Translational control in stem cells. Front Genet.

[CR70] Tamm C, Duckworth J, Hermanson O, Ceccatelli S (2006). High susceptibility of neural stem cells to methylmercury toxicity: effects on cell survival and neuronal differentiation. J Neurochem.

[CR71] Tamm C, Duckworth JK, Hermanson O, Ceccatelli S (2008). Methylmercury inhibits differentiation of rat neural stem cells via Notch signalling. NeuroReport.

[CR72] Teo AKK, Arnold SJ, Trotter MW (2011). Pluripotency factors regulate definitive endoderm specification through eomesodermin. Genes Dev.

[CR73] Teo AK, Ali Y, Wong KY (2012). Activin and BMP4 synergistically promote formation of definitive endoderm in human embryonic stem cells. Stem Cells.

[CR74] Theunissen PT, Pennings JL, Robinson JF, Claessen SM, Kleinjans JC, Piersma AH (2011). Time-response evaluation by transcriptomics of methylmercury effects on neural differentiation of murine embryonic stem cells. Toxicol Sci.

[CR75] Tsankov AM, Akopian V, Pop R (2015). A qPCR ScoreCard quantifies the differentiation potential of human pluripotent stem cells. Nat Biotechnol.

[CR76] Ung CY, Lam SH, Hlaing MM (2010). Mercury-induced hepatotoxicity in zebrafish: in vivo mechanistic insights from transcriptome analysis, phenotype anchoring and targeted gene expression validation. BMC Genomics.

[CR77] Van Oostdam J, Donaldson SG, Feeley M (2005). Human health implications of environmental contaminants in Arctic Canada: a review. Sci Total Environ.

[CR78] Visan A, Hayess K, Sittner D (2012). Neural differentiation of mouse embryonic stem cells as a tool to assess developmental neurotoxicity in vitro. Neurotoxicology.

[CR79] Woods JS, Ellis ME (1995). Up-regulation of glutathione synthesis in rat kidney by methyl mercury: relationship to mercury-induced oxidative stress. Biochem Pharmacol.

[CR95] Wu J, Ying T, Shen Z, Wang H (2014). Effect of low-level prenatal mercury exposure on neonate neurobehavioral development in China. Pediatr Neurol.

[CR80] Xuan S, Sussel L (2016). GATA4 and GATA6 regulate pancreatic endoderm identity through inhibition of hedgehog signaling. Development.

[CR81] Yadetie F, Karlsen OA, Lanzén A (2013). Global transcriptome analysis of Atlantic cod (Gadus morhua) liver after in vivo methylmercury exposure suggests effects on energy metabolism pathways. Aquat Toxicol.

[CR82] Yan P, Ren J, Zhang W, Qu J, Liu G-H (2020). Protein quality control of cell stemness. Cell Regener.

[CR83] Yang J, Jiang W (2020). The role of SMAD2/3 in human embryonic stem cells. Front Cell Dev Biol.

[CR84] Yang C-Y, Liu S-H, Su C-C (2022). Methylmercury induces mitochondria-and endoplasmic reticulum stress-dependent pancreatic β-cell apoptosis via an oxidative stress-mediated JNK signaling pathway. Int J Mol Sci.

[CR96] You C-H, Kim B-G, Jo E-M (2012). The relationship between the fish consumption and blood total/methyl-mercury concentration of costal area in Korea. Neurotoxicology.

[CR85] Yu G, Wang L-G, Han Y, He Q-Y (2012). clusterProfiler: an R package for comparing biological themes among gene clusters. Omics: J Integr Biol.

[CR86] Zalups RK (2000). Molecular interactions with mercury in the kidney. Pharmacol Rev.

[CR87] Zimmer B, Schildknecht S, Kuegler PB, Tanavde V, Kadereit S, Leist M (2011). Sensitivity of dopaminergic neuron differentiation from stem cells to chronic low-dose methylmercury exposure. Toxicol Sci.

