# Supplementary material for: Effects of low doses of methylmercury (MeHg) exposure on definitive endoderm cell differentiation in human embryonic stem cells
Source: Arch Toxicol. 2023 Aug 23;97(10):2625–41. doi: 10.1007/s00204-023-03580-7 (PMC10475006; doi:10.1007/s00204-023-03580-7)
Supplement: Supplementary file 5 — Supplementary file5 (DOCX 1424 KB) [file 204_2023_3580_MOESM5_ESM.docx]

Table S1. Primer information for qPCR

| Gene name | Accession No./Reference | Sequence |
| --- | --- | --- |
| URB1 | NM_014825.3 | F: 5’-CATCTGCCCGGACCCCATAG-3’  R: 3’-TCCTGACAAAGCACTTCCTCTC-5’ |
| SMAD3 | PMID: 25140399 | F: 5’-CCCCAGCACATAATAACTTGG-3’  R: 3’-AGGAGATGGAGCACCAGAAG-5’ |
| BMP4 | NM_001202.6 | F: 5’-GGGATTCCCGTCCAAGCTAT-3’  R: 3’-ACGGAATGGCTCCATAGGTC-5’ |
| GATA6 | NM_005257.6 | F: 5’-AGAAGCGCGTGCCTTCATC-3’  R: 3'-ATAGCAAGTGGTCTGGGCAC-5’ |
| EOMES | PMID: 29382828 | F: 5’-CGGCCTCTGTGGCTCAAA-3’  R: 3’-AAGGAAACATGCGCCTGC-5’ |
| FOXA2 | NM_021784.5 | F: 5’-TGCACTCGGCTTCCAGTATG-3’  R: 3’-CGTGTTCATGCCGTTCATCC-5’ |
| GAPDH | NM_002046.7 | F: 5’-TCGGAGTCAACGGATTTGGT-3’  R: 3’-TTCCCGTTCTCAGAATTGAC-5’ |
| HDDC2 | NM_016063.3 | F: 5’-GAAGCGGTCATGAAAGTGCC-3’  R: 3’-ATGAACCTAGCCTTCGGGGA-5’ |
| ZNF324B | PMID: 30927132 | F: 5’-CATTGGAAGGACAAACCTAGGATGATG-3’  R: 3’-CTTATCTGCTCCAAAGCTATCACTGTC-5’ |

Table S2. Antibody information used for Western Blot

| Marker | Dilution | Catalog No. | Company |
| --- | --- | --- | --- |
| BHMT | 1:1000 in NFDM | PA5-21466 | Thermo Fisher Scientific |
| URB1 | 1:500 in NFDM | 20023-1-AP | Thermo Fisher Scientific |
| SMAD | 1:1000 in NFDM | MA5-14939 | Thermo Fisher Scientific |
| p-SMAD | 1:500 in BSA | ab52903 | Abcam |
| BMP4 | 1:500 in BSA | MA5-15572 | Thermo Fisher Scientific |
| GATA6 | 1:1000 in NFDM | PA5-40559 | Thermo Fisher Scientific |
| EOMES | 1:500 in NFDM | MA5-24291 | Thermo Fisher Scientific |
| LEFTY2 | 1:1000 in NFDM | PA5-21367 | Thermo Fisher Scientific |
| FOXA2 | 1:1000 in NFDM | ab108422 | Abcam |
| PSMB3 | 1:1000 in NFDM | PA5-28999 | Thermo Fisher Scientific |
| LC3B | 1:1000 in NFDM | PA5-32254 | Thermo Fisher Scientific |
| SQSTM1/p62 | 1:1000 in NFDM | ab56416 | Abcam |
| Goat anti-Rabbit secondary antibody, HRP | 1:5000 in BSA/NFDM | G-21234 | Thermo Fisher Scientific |
| Goat anti-Mouse secondary antibody, HRP | 1:5000 in BSA/NFDM | ab97023 | Abcam |


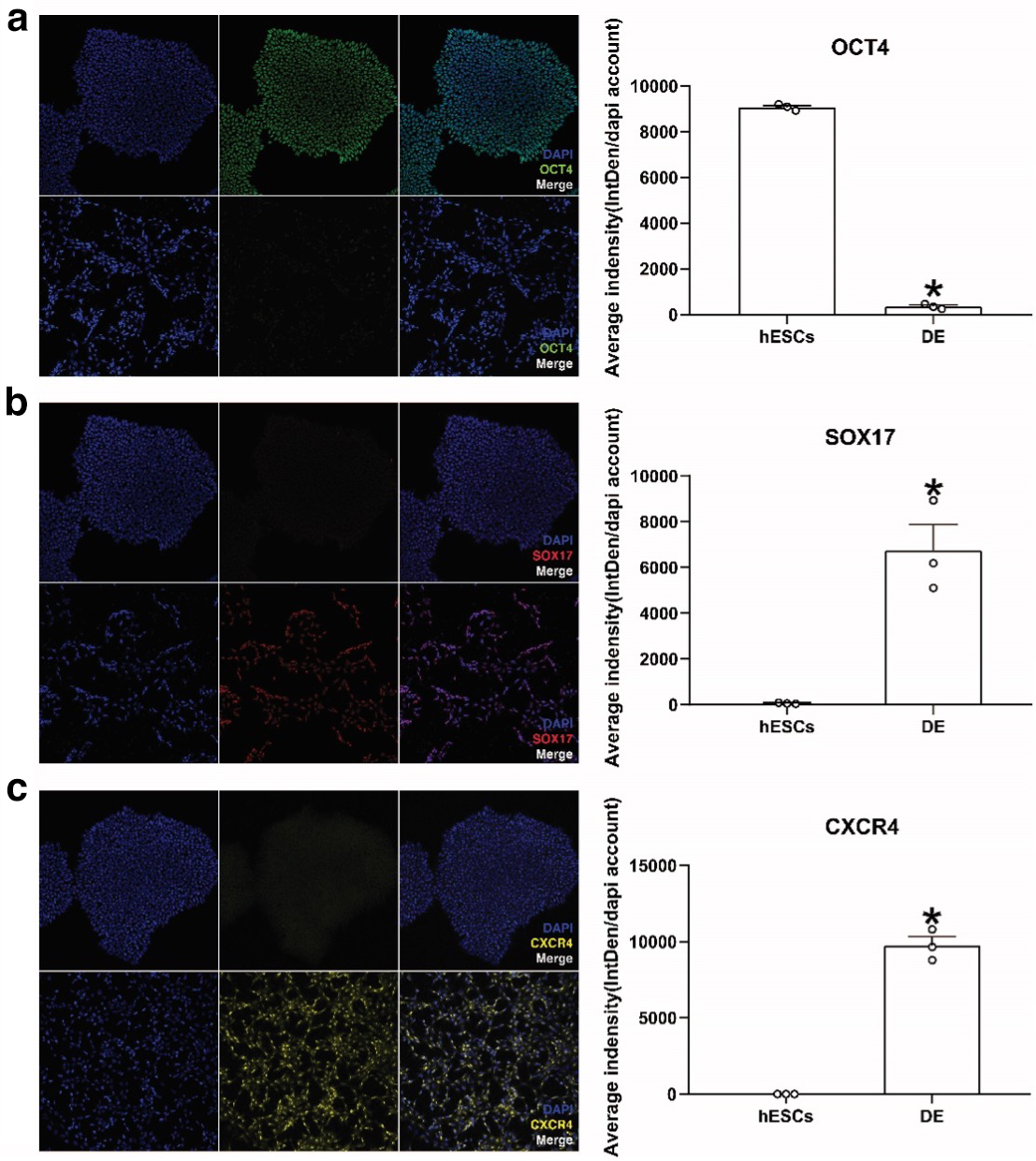


Fig. S1 Verification of DE induction before conducting experiments. Pre-induction hESCs and induced DE cells were immunostained for both pluripotency marker (OCT4) and definitive endoderm markers (SOX17 and CXCR4). Green indicates positive staining of OCT4 (a). Red indicates positive staining of SOX17 (b). Yellow indicates positive staining of CXCR4 (c). Blue indicates positive staining of nuclei using DAPI. The fluorescent intensity was normalized to DAPI numbers. Data are presented as means ± SEMs with N = 3 for each group. The small circle symbols represent individual values of each experiment. *T-test* was conducted to compare differences between the average fluorescent intensity of hESCs and DE cells with p < 0.05 being considered as significant marked by asterisk


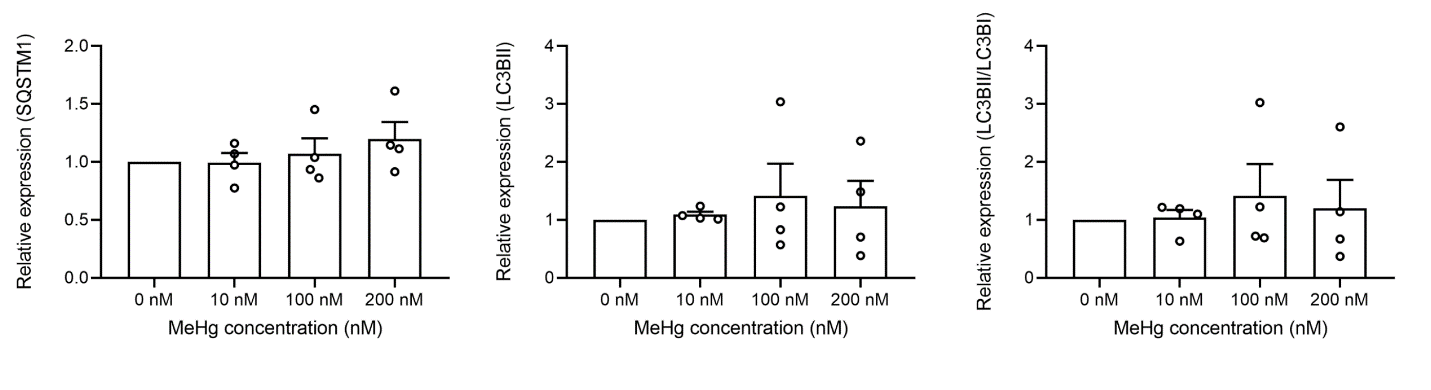


Fig. S2 Expression of autophagy related proteins SQSTM1, LC3BII and ratio of LC3BII/LC3BI in definitive endoderm (DE) cells exposed to MeHg during human embryonic stem cells to DE cell differentiation. Data are presented as means ± SEMs with N = 4 for each protein. Each batch was first normalized to 0 nM exposure group before comparison. The small circle symbols represent individual values of each experiment. One-way ANOVA and Dunnett’s post hoc test were used at each time point to compare differences between treatment and control groups with p < 0.05 being considered as significant marked by asterisk
